# Time since last drink is positively associated with heart rate variability in outpatients with alcohol use disorder: Further evidence of psychophysiological recovery in early alcohol use disorder recovery

**DOI:** 10.21203/rs.3.rs-2986948/v1

**Published:** 2023-06-02

**Authors:** David Eddie, Agata Pietrzak, Jason Ham

**Affiliations:** Massachusetts General Hospital; Massachusetts General Hospital; Boston University

**Keywords:** alcohol use disorder, alcohol abstinence, psychophysiological recovery, heart rate variability

## Abstract

Heart rate variability (HRV) is a biomarker of psychological and physiological health with greater variability reflecting greater psychophysiological regulatory capacity. The damaging effects of chronic, heavy alcohol use on HRV have been well explored, with greater alcohol use associated with lower resting HRV. In this study we sought to replicate and extend our previous finding that HRV improves as individuals with alcohol use disorder (AUD) reduce or stop drinking and engage in treatment. With a sample of treatment engaged adults in the first year of a current AUD recovery attempt (*N* = 42), we used general linear models were used to explore associations between indices of HRV (dependent variables) and time since last alcoholic drink at study baseline assessed using timeline follow-back (independent variable), with checks for effects of age, medication, and baseline AUD severity. As predicted, HRV increased as a function of time since last drink, however, contrary to hypotheses, HR did not decrease. Effect sizes were largest for HRV indices fully under parasympathetic control, and these significant associations remained after controlling for age, medications, and AUD severity. Because HRV is an indicant of psychophysiological health, as well as self-regulatory capacity that may portend subsequent relapse risk, assessing HRV in individuals entering AUD treatment could provide important information about patient risk. At-risk patients may do well with additional support and may especially benefit from interventions like Heart Rate Variability Biofeedback that exercise the psychophysiological systems regulating brain/cardiovascular communication.

## INTRODUCTION

Heart rate variability (HRV)—the variance in heartbeat intervals—is a biomarker of physiological and psychological health with greater variability reflecting greater physiological and psychological flexibility and regulatory capacity (Eddie et al., 2021). Conversely, lower HRV is commonly observed in people experiencing disease states (Berntson et al., 2008), psychological disorders (Beauchaine & Thayer, 2015), and chronic stress (Kim et al., 2018).

The effects of chronic, heavy alcohol use on HRV have been well explored, with greater alcohol use associated with lower resting HRV (Quintana et al., 2013). It has been suggested that HRV reductions in individuals engaged in chronic, heavy alcohol use is both a result of the direct pharmacological effects of drugs on the cardiovascular system, as well as a reflection of loss of top-down psychophysiological control associated with substance use disorders (SUD; Bates & Buckman, 2013; Eddie et al., 2022).

Thankfully, the systems regulating HRV are usually capable of recovery. We have previously observed that women receiving Cognitive Behavioral Therapy for alcohol use disorder (AUD) demonstrate increased resting, laboratory assessed HRV from the start to end of treatment (Buckman et al., 2019). This effect remained even after accounting for any drinking during treatment, suggesting that AUD treatment may confer physical health benefits in addition to any physiological benefits associated with reducing or stopping drinking.

This is an important finding because HRV is an indicant of self-regulatory capacity and HRV recovery likely impacts one’s capacity to achieve AUD remission. For instance, we recently showed in the present sample that greater HRV predicted more percent days abstinent (i.e., less drinking) over 90-day follow-up (Eddie et al., Under review). If HRV does indeed improve on average in early AUD recovery, it could provide a normative, objective indicant of treatment response to complement existing self-report measures that track HRV might ultimately be utilized as an objective indicant of recovery capacity or risk. For instance, HRV could be regularly assessed with usual subjective measures of treatment response.

In this study, we sought to replicate our previous findings by exploring the relationship between time since last alcohol use and HRV among individuals in the first year of a current AUD recovery attempt undergoing HRV monitoring. This study extends past research in that it utilized a mixed-sex sample, and HRV was measured under real-world conditions, rather than at rest in a laboratory. Based on previous findings, we hypothesized that greater time since last alcohol use would be associated with greater HRV and lower heart rate (HR). Checks were included to explore potential covarying effects of age, medication, and baseline AUD severity.

## METHODS

Detailed methods can be found in Eddie et al. (Under review). Briefly, participants were adults 18 + meeting DSM-5 diagnostic criteria for past-year AUD who were in the first year of a current AUD recovery attempt and participating in outpatient AUD treatment or AUD mutual-help programs like Alcoholics Anonymous or SMART Recovery. Individuals taking medications that directly affect HRV like beta-blockers were excluded from the study, however, other medications were allowed.

At study intake, participants completed baseline measures and reported how many days prior their last use of alcohol had been. Participants were then fitted with an eMotion Faros 180 ambulatory electrocardiogram (ECG) monitor, which they wore for four days. Participants were asked to wear the device throughout the day without changing their normal behavior. ECG was sampled at 250Hz.

This study was approved by the institutional review board at Mass General Brigham (IRB# 2016P001178).

## MEASURES

### Time since last substance use

Time since last substance use was assessed with the question, “How many days ago was your last drink?” If participants had difficulty recalling exactly how many days ago their last drink was, the experimenter helped them determine this with the assistance of a calendar.

### Heart rate variability measures

Detailed ECG processing methods can be found in Eddie et al. (Under review). HRV statistics were calculated for 5-minute epochs of ambulatory ECG recording aligning with ecological momentary assessment surveys (not included in this study). On average, there were 10.00 (*SD* = 3.94; range = 2–21) epochs of ECG recording utilized per participant.

Study indices included heart rate (HR), the standard deviation of normal-normal intervals (SDNN), root mean squared of successive differences (RMSSD), the percent of adjacent normal-normal intervals differing by greater than 50 milliseconds (pNN50), high frequency HRV (HF HRV; 0.15–0.4Hz), and low frequency HRV (LF HRV; 0.04–0.15Hz).

The HRV indices RMSSD, pNN50, and HF HRV reflect parasympathetic control of the heart, while SDNN reflects overall HRV and likely includes aspects of both parasympathetic and sympathetic control (Malik et al., 1996). Greater scores on these indices, and lower HR, are indicative of greater parasympathetic activation. LF HRV reflects parasympathetic control with varying amounts of sympathetic and baroreflex influence depending on conditions (Goldstein et al., 2011; Reyes del Paso et al., 2013; Thayer et al., 2010).

### Control measures

Because factors that commonly covary with HRV might have been influencing study findings, we checked for effects of: 1) age, which is typically negatively associated with HRV, 2) medications (yes/no), and 3) AUD severity (measured with the Alcohol Dependence Scale; Kivlahan et al., 1989), which is typically negatively associated with parasympathetically-driven HRV (Quintana et al., 2013).

## ANALYSES

In order to identify potential multivariate outliers, Mahalanobis distances were calculated with the variables HR, HF HRV, and LF HRV. HRV indices were then checked for normality; SDNN, RMSSD, pNN50, HF HRV, and LF HRV were found to be excessively skewed and kurtotic and were thus logarithmically transformed. Then, an average score for each index was calculated for each participant across all included recording epochs.

To explore associations between length of alcohol abstinence and HRV, we ran general linear models using the GLM procedure in SAS 9.4 (SAS Institute, 2023). Separate models were run for each HRV index, with HRV indices as the dependent variable, and length of alcohol abstinence as the independent variable. Effect sizes are represented with R-squared, with an effect size considered small if R^2^ = 0.01, medium if R^2^ = 0.09, and large if R^2^ = 0.25.

Lastly, to check if factors known to be associated with HRV could be influencing length of abstinence/HRV relationships, we added age, medication/s (yes/no), and AUD severity (measured with the Alcohol Dependence Scale; Kivlahan et al., 1989) to our multivariate models to explore how these factors influenced results.

## RESULTS

The sample (*N* = 42) was 38.10% female and 61.90% male with ages ranging from 18–65 (*M* = 41.59, *SD* = 12.60). Participants identified as White/European American (73.81%), Black/African American (19.05%), Asian (4.76%), and Other race/Mixed race (2.38%). Physiology data were missing for two participants (one due to ECG device failure and one due to a lost ECG device) leaving a total of *n* = 40 included in the present analysis. Multivariate outlier testing identified two participant epochs as outliers, which were not included in the analyses.

### Descriptive statistics

Baseline assessment indicated the sample had, on average, severe AUD, with a mean Alcohol Dependence Scale score of 23.80 (*SD* = 8.49). The sample had an average Beck Depression Inventory II (Beck et al., 1996) score of 17.60 (*SD* = 12.08), indicating mild depression, and a mean State Anxiety Inventory (Ramanaiah et al., 1983) score of 32.78 (*SD* = 7.35), suggesting mild anxiety.

On average, at study baseline, participants reported 87.33 (*SD* = 101.29) days since their last alcohol use. Mean and standard deviations for study HRV statistics are presented in Table 1.

### General linear model results

Findings from the bivariate general linear models are reported in Table 1. As hypothesized, greater HRV indicated by higher SDNN, RMSSD, pNN50, HF HRV, and LF HRV was associated with greater time since last drink. Effect sizes for length of abstinence/HRV relationships were medium to large. However, contrary to hypotheses, HR was not significantly associated with time since last drink.

Adding age, medication/s (yes/no), and AUD severity to the HR, RMSSD, pNN50, and HF HRV models did not markedly alter their results. However, associations between SDNN and LF HRV, and time since last alcohol use were no longer statistically significant after adding these covariates to the respective models (*p* > .05).

## DISCUSSION

We have previously shown that women with AUD show improvements in HRV while AUD treatment (Buckman et al., 2019). The present study sought to replicate and extend this previous work by exploring associations between length of alcohol abstinence and HRV using ambulatory ECG monitoring in a mixed sex sample of individuals in the first year of a current AUD recovery attempt. As hypothesized, HRV increased as a function of time since last drink assessed at study baseline, however, contrary to hypotheses, HR did not decrease. Effect sizes were largest for HRV indices fully under parasympathetic control (i.e., RMSSD, pNN50, HF HRV), and these significant associations remained after controlling for age, medications, and AUD severity. Abstinence/HRV relationships for the indices SDNN and LF HRV were less robust, as indicated by smaller effect sizes, and notably, after controlling for age, medications, and AUD severity, these associations were no longer statistically significant.

These findings are consistent with our previous work showing larger effect sizes for parasympathetic HRV recovery in individuals in treatment for AUD, versus overall HRV (Buckman et al., 2019). Given the impact of chronic, heavy alcohol use on resting state parasympathetically mediated HRV (Cheng et al., 2019), it is perhaps not surprising that parasympathetic HRV measures like RMSSD, pNN50, and HF HRV rebound most when drinking is reduced or stopped and individuals engage with treatment.

This observed HRV recovery is prognostically important given we have also shown with these data that HRV can predicted subsequent alcohol use over 90-day follow-up (Eddie et al., Under review). This suggests HRV recovery is not just an indicant of normalization of psychophysiological functioning, but that HRV is also is an indicant of self-regulatory capacity portending subsequent AUD related behaviors. In this way, HRV may be viewed as a marker of physiological and psychophysiological health and recovery, as well as a marker of prognostic risk.

There is an important clinical implication here. If additional studies replicate the positive association between time since last alcohol use and HRV it might be expected that individuals entering treatment for AUD with a short latency of alcohol use would have lower than average HRV. Psychophysiological recovery through the course of treatment would be indicated by increasing HRV levels at subsequent assessment and would portend better recovery outcomes. On the other hand, individuals entering treatment with greater latency of alcohol use but low HRV, or individuals with low HRV at treatment initiation who do not demonstrate increases in HRV through treatment may be at particular risk and need additional support.

Such individuals may especially benefit from Heart Rate Variability Biofeedback (Lehrer et al., 2000), a breathing-based intervention that exercises the psychophysiological systems regulating brain/cardiovascular communication that has been shown to reduce craving and other forms of negative affect in individuals with substance use disorders (Eddie et al., 2021; Leyro et al., 2019). This intervention might be particularly advantageous for such individuals because it targets a vulnerability not directly addressed by first-line substance use disorder intervention like Cognitive Behavioral Therapy and mutual-help programs.

### Limitations

This study was not without limitations, which included: 1) a small sample size, 2) alcohol use was not assessed during the four-day ECG monitoring period, and 3) the analytic approach included multiple comparisons that increased the chance of type-II error. These limitations should be considered with the study’s strengths, which included high ecological validity associated with the use of *in natura* ECG monitoring and a naturalistic sample of individuals seeking recovery from AUD.

## CONCLUSIONS

The present study replicates and extends our previous finding that HRV increases among people with AUD as a function of reducing or stopping drinking and engaging in treatment. Because HRV is an indicant of psychophysiological health, as well as self-regulatory capacity that may portend subsequent relapse risk, assessing HRV in individuals entering AUD treatment could provide important information about patient risk. At-risk patients may do well with additional support, and may especially benefit from Heart Rate Variability Biofeedback, a breathing-based intervention that exercises the psychophysiological systems regulating brain/cardiovascular communication.

## Figures and Tables

**Table 1.** Average heart rate (HR) and heart rate variability (HRV) with standard deviations (left column), and unadjusted, general linear model results with HRV index as dependent variable, and time since last alcoholic drink as independent variable.

	Mean (*SD*)		Model		Follow-up PDA	
		*df*	*F*	*R* ^2^	*β*	*SE*
Heart Rate (bpm)	89.87 (17.10)	1,38	2.32	0.06	−0.030	0.017
SDNN (ms) ^a^	54.86 (48.43)	1,38	7.63**	0.17	0.001**	0.001
RMSSD (ms) ^a^	22.57 (19.42)	1,38	11 22**	0.23	0.003**	0.001
pNN50 (%) ^a^	6.36 (9.42)	1,38	11.84**	0.24	0.005**	0.001
HF HRV (ms^2) ^a^	272.86 (614.74)	1,38	9.45**	0.20	0.005**	0.002
LF HRV (ms^2) ^a^	664.57 (859.43)	1,38	7.96**	0.17	0.004**	0.001

***Notes.** SD*= standard deviation (in parentheses); bpm= beats per minute; ms= milliseconds; SDNN= standard deviation of all normal-to-normal intervals; RMSSD= root of the mean squared differences of successive normal-to-normal intervals; pNN50= percent of normal-to-normal adjacent intervals greater than 50ms; HF HRV= high frequency heart rate variability; LF HRV= low frequency heart rate variability.

a Logarithmically transformed data used for analyses; untransformed means are presented in first column.

*df*= degrees of freedom; *β*= unstandardized beta coefficient; *SE*= standard error.

* *p*< .05

** *p*< .01.
